# Inferring Contacting Residues within and between Proteins: What Do the Probabilities Mean?

**DOI:** 10.1371/journal.pcbi.1004726

**Published:** 2016-05-12

**Authors:** Erik van Nimwegen

**Affiliations:** Biozentrum, University of Basel, Basel, Switzerland; Politecnico di Torino, ITALY

Although correlations between residues occurring at different positions within multiple protein alignments have long been used to infer directly interacting residues, most of these methods suffer from the frequent occurrence of indirectly correlated residues. Recently, a number of methods have been proposed that incorporate the entire set of observed frequencies of amino acid pairs for all pairs of positions into rigorous probabilistic models; these models have been shown to strongly improve the prediction of directly interacting residues by successfully distinguishing direct from indirect correlations. Some of these methods make use of the maximum entropy principle, originating in statistical physics. It has recently been argued that there is no reason to assume that protein sequences should follow maximum entropy distributions and that it is therefore puzzling that the max-ent formalism is successful for predicting interacting residues in proteins. In this brief review I will argue that such apparent puzzles result from a misconception of the meaning of the max-ent formalism and, more generally, on the meaning of probabilities.

## The Unreasonable Effectiveness of Max-ent Distributions

The project I was given at the start of my PhD research involved developing a theory for the dynamics of populations evolving on a particular toy fitness landscape. My advisers suggested that, instead of modeling the whole distribution of genotypes in the population, I would try to find an effective low-dimensional description in terms of fitness classes. Having been trained in theoretical physics, it seemed natural to me to use the maximum entropy (max-ent) formalism to derive the effective dynamics of these “macroscopic” variables from the underlying “microscopic” dynamics of genotypes, and we were pleased to find that this approach led to excellent predictions [1]. Naturally, we next aimed to prove that the max-ent approach was “correct” for this system, i.e., that the “true” distribution of the population in genotype space was well approximated by the max-ent distribution. However, some calculations quickly showed this not to be the case. Whereas the max-ent approach effectively assumed that populations would spread uniformly over iso-fitness subspaces in genotype space, one can show that under mutation and selection, populations distribute themselves highly non-uniformly in a manner that depends on the topology of the iso-fitness subspace [2]. This raised a puzzle: If the max-ent distribution was clearly “wrong,” why then was it successfully predicting the macroscopic dynamics?

Ever since the max-ent formalism was introduced, primarily by Gibbs [3], this question has cropped up repeatedly in different contexts; we have lots of evidence suggesting the max-ent distribution cannot possibly match the “true” distribution of our system, but its predictions are highly successful. It was recently raised again by Erik Aurell [4] in an interesting discussion among the participants of the “Regulation and Inference in Biological Networks” meeting at Bardonecchia, Italy, this time in the context of the recent success in predicting 3-D contacts in protein structures from sequence alignments using max-ent methods [5–8], which have been successfully used to predict protein folds [8–11]. That is, why would max-ent approaches such as the direct coupling analysis (DCA) perform so well on predicting protein contacts when there is no reason to assume the “true” distribution of sequences that share a common structure follows this max-ent distribution? In this article, I would like to share my views on the general question of the justification for max-ent approaches and the application to predicting interactions among residues in proteins in particular.

To return to my own first encounter with this question, fate had it that, not long after I convinced myself that the max-ent assumption must be wrong for the evolutionary system I was studying, I moved to another desk and found that a previous occupant had left a large pile of printouts in a drawer carrying the title “Probability Theory, the Logic of Science” [12]. In retrospect, I feel that finding this printout of E. T. Jaynes’ seminal work on probability theory was one of the most important events in my scientific career. The philosopher Ludwig Wittgenstein has suggested that philosophical questions are solved not by finding an answer, but rather by developing a point of view that makes them simply evaporate. Reading Jaynes not only cleared up my max-ent puzzle, it evaporated all conceptual problems that were previously nagging me regarding probability theory, statistics, and statistical physics, leaving me with a profound understanding of just how general techniques like max-ent can be applied. The key to making these conceptual problems evaporate was to fundamentally rethink what a probability is.

## What Are Probabilities?

The terminology of the traditional statistics and probability theory of the 20th century is completely suffused with the idea that probabilities are real, measurable properties of physical events. In this view, the physical world contains both variables with definite values, as well as “random variables” that are indefinite and subject to chance/randomness, with some true “distribution” of probabilities that can be measured by repeated experiments or trials. The probability for an experiment to come out a certain way is imagined to be determined by “nature.” Although such a viewpoint is a reasonable working model within a narrow range of applications, it is conceptually misleading and has been the source of countless misunderstandings over the last century.

First, to anyone familiar with basic physics, this interpretation of probabilities is completely absurd for the archetypical examples of “random variables” such as coin tosses, card shufflings, et cetera. Coin tosses are governed by Newtonian mechanics and their outcome is a deterministic function of the initial momentum and angular momentum of the coin. It is straightforward to construct machines that reliably toss a coin to come out a given way, and even humans can learn to toss coins to come out in any way they want e.g., here [13] one can see Persi Diaconis demonstrate this ability while explaining his views on what probabilities are. The reason that, in typical situations, one doesn’t know in advance how a coin toss is going to come out is because one doesn’t know the initial conditions of the toss. If different people have different information about these initial conditions, the probabilities are going to be different for them. When Persi Diaconis throws a coin, he can be virtually certain of its outcome, whereas you and I have no idea how he is going to toss it. The crucial point is that the probability is not an inherent property of a natural process. Rather, it is a property of a particular state of knowledge or information about a natural process.

As discussed by Jaynes [12], this viewpoint can be made mathematically rigorous, and what emerges is a compelling argument that probability theory is in fact a general calculus for reasoning with incomplete information. There are fairly strong mathematical arguments that, if one asks for a calculus that extends propositional logic to consider not only statements that are either true or false, but statements that can have an arbitrary degree of plausibility between zero (= false) and one (= true), then the solution is essentially unique and corresponds precisely to the probability calculus with which we are familiar [14,15]. In this interpretation, a probability represents a state of information. Jaynes formalizes this idea in a manner very similar to the way Turing formalized what a computation is by introducing a Turing machine [16]. That is, Jaynes imagines a machine that is fed with specific and rigorously defined pieces of information, which then uses this information to assign probabilities to statements. This nicely conveys the fact that probabilities are “subjective” in the sense that they are determined not by nature, but by a state of information, while at the same time removing all the connotations with taste and human psychology that the word “subjective” carries. The question that probability theory answers is: if a machine is specified these pieces of information, what probabilities should it assign?

## The Max-ent Formalism

To give an example, assume all the information we have regarding a variable *X* is that it can take on *n* possible states or values. If this is really all the available information, then nothing distinguishes the *n* possibilities from each other and the symmetry of this situation forces the machine to assign an equal probability 1/*n* to each possibility. That is, the information specified is invariant under arbitrary permutation of the *n* possibilities and the uniform distribution is the only distribution that respects this symmetry. The claim is not that all *n* possibilities are going to occur equally frequently. The claim is simply that the uniform distribution is the only one that correctly represents the information specified. Importantly, this interpretation of probability as representing a state of information does not preclude that, in some situations, probabilities can match frequencies. For example, if we now provide the machine with the additional information that, in *M* repeated trials, each possible outcome *i* occurred *m*_*i*_ times, then the probabilities that the machine will assign to the next trial coming out *i* will be *p*_*i*_ = (*m*_*i*_+1)/(*M*+*n*), which converges to the frequency of occurrence of *i* as *M* gets large. That is, all the results based on the traditional frequency interpretation of probability are still contained in this theory. But instead of the correspondence between probability and frequency being a postulate, this correspondence can be *derived* to occur in particular settings.

These kinds of symmetry arguments can be applied much more generally to calculate how to assign probabilities. The maximum entropy principle is just one of the tools in the toolbox for turning a state of information into a probability assignment. Specifically, if we have some system that can take on states *s* and we specify the information that certain functions *f*_1_(*s*),*f*_2_(*s*),…,*f*_*n*_(*s*) take on averages *f*_1_,*f*_2_,…,*f*_*n*_, then one can derive that the probability distribution that represents this state of information is the distribution *P*(*s*) that maximizes the entropy $H=-\sum_{s}P(s)\text{log}[P(s)]$ subject to the constraints. This distribution takes on the general form $P(s)\propto e^{-\lambda_{1}f_{1}(s)-\lambda_{2}f_{2}(s)-\cdots \lambda_{n}f_{n}(s)}$, where the constants *λ*_*i*_ (typically called Lagrange multipliers) are chosen such that the constraints 〈*f*_*i*_(*s*)〉 = *f*_*i*_ are all satisfied. This is all there is to the max-ent formalism. It does not claim that the distribution *P*(*s*) corresponds to the “true” frequencies with which different states *s* occur in nature. All it provides is the correct way of representing a particular state of information.

So why is the distribution *P*(*s*) that maximizes the entropy the correct representation of the information specified by the constraints? Typically there are infinitely many distributions that satisfy the contraints. So why not some other distribution that also satisfies the constraints? Probably the most intuitive explanation is that, as first argued by Claude Shannon [17], the entropy $H=-\sum_{s}P(s)\text{log}[P(s)]$ measures how much uncertainty, lack of information, or “not knowing” is associated with the distribution *P*(*s*). Therefore, of all distributions obeying the constraints, the max-ent distribution effectively makes the fewest assumptions. Any other distribution would correspond to making additional assumptions beyond the information that was specified. Max-ent distributions can also be derived under a number of other measures of optimality or consistency (see, e.g., [18,19]). In my personal opinion, the most attractive viewpoint is to see max-ent not as a fundamental principle, but as an effective rule that emerges asymptotically from applying the general rules of probability theory in particular settings. Max-ent distributions naturally emerge if, instead of assuming the fundamental hypothesis space consists of the states *s*, one introduces a deeper hypothesis space *X* in which each state *s* corresponds to a subset of states *x* in *X*. In this setting *P*(*s*) equals the size of the subset *s*, i.e., *P*(*s*) = |*s*|/|*X*|. If the only information about the division of *X* into subsets *s* is that the resulting distribution *P*(*s*) should obey certain constraints $\sum_{s}f(s)P(s)=\bar{f}$, then in the limit of |*X*|→∞, the distribution *P*(*s*) almost surely becomes the max-ent distribution. That is, among all ways of partitioning *X* into subsets, the max-ent distribution can be realized in overwhelmingly more ways than any other distribution *P*(*s*). Jaynes refers to a version of this derivation as the “Wallis derivation” [12], but it has been presented in the literature in many different guises.

## Max-ent Distributions for Protein Families

Let’s consider a concrete example that stays close to the topic under discussion, i.e., how to infer protein structure from sequence alignments. Assume we are given a multiple alignment *S* of sequences that are known to fold into a common structure *x*. Using this information, we want to construct a probability distribution *P*(*s*|*x*) for the probability that a sequence that folds into structure *x* will have sequence *s*. Perhaps the simplest possible model is obtained when we only make use of the set of amino acid frequencies at each position of the alignment. That is, let *f* denote the set of amino acid frequencies, with $f_{\alpha }^{i}$ the observed frequency of letter *α* at position *i*. We then construct the max-ent distribution *P*(*s*|*f*) that satisfies the constraints $\sum_{s}\delta_{s_{i},\alpha }P(s|f)=f_{\alpha }^{i}$, with $\delta_{s_{i},\alpha }$ the Kronecker-delta function that is one when amino acid *s*_*i*_ equals *α*, and zero otherwise. This max-ent model, which takes the form $P(s|f)=\prod_{i}f_{s_{i}}^{i}$, is typically referred to as a position-specific weight matrix (PSWM) and such models are also used to model the distribution of DNA sequences that a given transcription factor recognizes [20–22]. As a technical aside, in practice our knowledge of the frequencies $f_{\alpha }^{i}$ is based on finite data and thus has only limited accuracy, which leads to finite size corrections in a rigorous probabilistic calculation (see, e.g., [22]).

One may wonder whether the extremely simplistic PSWM model can have any use at all in predicting protein structure from sequence. But in fact, it lies at the core of de novo annotation of protein sequences from sequenced genomes. To do this, the elementary PSWM model is typically extended to allow for sequences of different lengths, by allowing some insertions and deletions; these extended models are known as HMM profile models [23]. Such HMM profiles have now been constructed for thousands of protein families (see, e.g., [24]). By applying these HMM models to naturally occurring sequences of protein-coding genes, new members of protein families can be successfully identified. Moreover, the multiple sequence alignments of protein families that are used as input for the algorithms that predict contacts are typically produced by aligning family members to such HMM profiles.

Clearly these simple PSWM models have proven extremely successful. At the same time, I doubt that there is any researcher who would take the max-ent distribution *P*(*s*|*f*) to be a realistic model of the “true distribution” *P*(*s*) of all sequences belonging to the family (leaving aside for the moment the question of how to even define such a “true” distribution *P*(*s*)). Intuitively, the reason that the simplistic model *P*(*s*|*f*) is successful in identifying additional members of the protein family is that the space of all possible sequences is so much larger than the subspace of sequences that have high probability under the model *P*(*s*|*f*) that, even though this is a very crude model, a naturally occurring protein sequence with high *P*(*s*|*f*) is very likely to be another member of the family. More generally, the key observation is that, independent of what the “true distribution” *P*(*s*) is, the amino acid frequencies $f_{\alpha }^{i}$ are enough to reliably identify which naturally occurring protein sequences come from the same family. Of course, the fact that the frequencies $f_{\alpha }^{i}$ are enough to detect additional members of the protein family does not mean this information suffices to answer any question about the protein family. For example, it is not sufficient for constructing synthetic protein sequences that fold into the same structure. That is, if we randomly sample sequences from *P*(*s*|*f*), these sequences are very unlikely to fold into the same structure [25]. Success or failure of the max-ent distribution on a particular problem has little to do with whether it accurately describes a “true” distribution, but rather reflects to what extent the provided information suffices for solving the problem at hand. Once one grasps this point, it becomes unnecessary to speculate about the true underlying distribution *P*(*s*). All one ever has is limited information about *P*(*s*) and methods for calculating the implications of this information. Whether a true underlying distribution *P*(*s*) exists or not becomes irrelevant and questions about success of max-ent methods should start to evaporate.

Because the amino acids at different positions are completely independent in the PSWM model *P*(*s*|*f*), this model is obviously incapable of making any predictions about interactions between amino acids. Thus, when the main interest is in predicting interactions between amino acids, arguably the simplest models are those that use the set of pairwise statistics $\left\{f_{\alpha \beta }^{ij}\right\}$. There is a considerable history to the idea of using “correlations” between the occurrence or evolution of amino acids at pairs of positions to infer interactions (see [26] for a recent review). However, the current discussion is precipitated by recent methods that achieved significant improvements in predicting interactions due to their ability to distinguish direct from indirect dependencies by rigorously modeling the entire set of pairwise frequencies $\left\{f_{\alpha \beta }^{ij}\right\}$. Lapedes et al. [27] provided the first theoretical discussion of how direct dependencies may be inferred from the set of pairwise statistics using max-ent, but did not develop a practical method. As far as I am aware, the first example of a practical method for rigorously incorporating all pairwise frequencies $\left\{f_{\alpha \beta }^{ij}\right\}$ to predict protein—protein interactions was presented in [28], and the same Bayesian network model was later applied to the problem of predicting contacts within proteins [29]. A first practical implementation of the max-ent approach to this problem, typically referred to as direct coupling analysis, was developed by Weigt et al. [5] in the context of the same two-component system problem, and a number of technical improvements have been reported since (see, e.g., [6–8,30,31]). The extent to which these models have been successful simply implies that the information contained in the pair statistics is sufficient for inferring protein contacts. This again does not imply that this information suffices for answering any question about the protein family. For example, an interesting question is whether the pair-statistics $\left\{f_{\alpha \beta }^{ij}\right\}$ are sufficient to design new sequences that fold into the same structure, i.e., whether synthetic sequences drawn from the max-ent distribution $P(s|\{f_{\alpha \beta }^{ij}\})$ are likely to fold into the target structure. As I mentioned already, sequences drawn from the PSWM distribution *P*(*s*|*f*) are highly unlikely to fold into the desired structure, but there are some results suggesting that sequences drawn from the distribution $P(s|\{f_{\alpha \beta }^{ij}\})$ that incorporates the pair statistics are more likely to fold into the structure, at least for one example protein domain [25].

## The Value of Incorrect Predictions

What if the max-ent distribution conditioned on some information *I* makes definite predictions that turn out to be wrong? This then implies that either some of the assumptions contained in information *I* are incorrect, or there are additional constraints (not contained in *I*) that significantly affect the predictions. Of course, such things do occur in practice and some have argued that this invalidates the max-ent approach, i.e., because there is no guarantee the predictions will turn out to be correct. However, the cases where max-ent predictions fail to match experimental results are often precisely the most informative ones. For example, the birth of quantum theory in physics essentially resulted from the realization by Planck, Einstein, and others that in order for predictions of statistical mechanics to match experimental observations, one had to assume certain variables had to be quantized, e.g., the energy *E* of radiation at frequency *v* emitted by a black body is constrained to be an integer multiple of *hv*, with *h* Planck’s constant. Similarly, in the contact prediction problem it has been observed that stretches of consecutive gap positions often occur in a highly correlated manner and incorporating such additional constraints improves contact prediction [31]. In other cases “false predictions” may actually point to errors in our underlying assumptions. For example, in some cases one finds that pairs of residues (*i*, *j*) that were strongly predicted to interact directly were not contacting in the protein structure, but were instead directly interacting in a homodimer or an alternative conformation of the protein [7].

It is sometimes argued that, if one interprets max-ent as working out the consequences of a specific state of information, then it is fundamentally flawed to use only some of our information, such as the pair frequencies $\left\{f_{\alpha \beta }^{ij}\right\}$, because our “state of information” includes many more things than these pair frequencies, e.g., we have the full multiple sequence alignment *S*. However, there is a virtually infinite list of facts that we could conceivably consider to include in our “state of information”: not only what all sequences in *S* are, but also what organisms they derived from, when the sequences were submitted to Genbank, by whom, and whether that person was wearing a yellow sweater at the time. There are countless facts that are obviously irrelevant to whatever we want to predict, some facts that are obviously crucial, and a large grey zone of information, the relevance of which is unclear. Whenever we build a model, we must make a choice of what information *I* to incorporate, including all assumptions that the model presupposes. In fact, one might argue that the whole enterprise of science revolves around finding out what information *I* about a system *X* is crucial for accurately predicting some property of interest *Y*. To the extent that we have not yet formalized all possible ways of formulating hypotheses and building models, this is still a necessarily subjective process. However, whereas the process of choosing what model to build and what information to incorporate may be subjective, the progress of science is helped enormously by the fact that we are now in possession of an objective mathematical methodology for rigorously calculating the predictions implied by information *I*, and only information *I*. As I have tried to argue in this review, this is precisely what is provided by the max-ent formalism and more generally by probability theory viewed as extended logic.

## Theoretically Optimal Contact Prediction from Sequence Alignments?

Finally, going beyond conceptual issues, it may be worthwhile to note that, at least formally, probability theory uniquely specifies how to assign probabilities to contacts given only a sequence alignment as information and to compare this “formal solution” with the Bayesian network and DCA approaches that have been put forward. Let *S* again denote an alignment of sequences known to fold into a common structure and let *G* denote a hypothesized contact graph for this structure. The contact inference problem formally consists in determining probabilities *P*(*S*|*G*,*I*) of the observed sequence alignment *S* for each possible contact graph *G* and whatever additional information *I* we wish to assume. Of course, in the real world we have all kinds of information, such as the laws of physics and chemistry, but here we are interested in calculating *P*(*S*|*G*,*I*) using only the dependency structure implied by the contact graph *G* and nothing else. Roughly speaking, the only information we assume is that the probability of each amino acid should only directly depend on its neighbors in the graph *G*. Such dependency notions are formalized by Markov random field models [32] and by the Hammersley-Clifford theorem (also known as the fundamental theorem of Markov random fields) [33], which states any positive probability distribution consistent with dependency graph *G* takes on the general Gibbs-distribution form

$$P(s|\{E_{c}\},G)=\frac{1}{Z(\{E_{c}\})}\text{exp}\left[\sum_{c\in G}E_{c}(s_{c})\right]$$ (1)

where the sum is over all maximal cliques *c* of the graph *G* and the *E*_*c*_ are “potentials” that assign a real number to each possible combination of the letters *s*_*c*_ of clique *c*. Thus, given a contact graph *G*, any probability distribution satisfying the conditional (in)dependencies implied by *G* can be specified by specifying a set of potentials {*E*_*c*_}, which I will collectively denote by *E*.

To calculate the probability *P*(*S*|*G*), probability theory now tells us we have to marginalize over the unknown potentials *E*. That is, we assign a (uninformative) prior *P*(*E*|*G*) to the space of all possible potential functions, and then calculate the integrals

$$P(S|G)=\int dE\prod_{s\in S}P(s|E,G)P(E|G).$$ (2)

Using this likelihood *P*(*S*|*G*) together with a prior *P*(*G*) over possible graphs *G* (which might be uniform if we assume no prior information on possible contact graphs), one obtains the posteriors $P\left(G|S\right)=P\left(S|G\right)P\left(G\right)/[\sum_{G^{\prime }}P\left(S|G^{\prime }\right)P\left(G^{\prime }\right)]$. Finally, the probability for a particular pair of positions to contact is given by summing *P*(*G*|*S*) over all graphs *G* in which this contact occurs.

Unfortunately, although probability theory specifies that this is in principle the calculation one would want to perform, the integrals over all possible potential functions *E* do not not seem tractable and neither is the sum over all possible contact graphs *G*. We are thus forced to make approximations. The Bayesian network model mentioned above provides a straightforward approximation by making the strong simplifying assumption that the graph *G* can only a be a tree. Consequently, the distribution *P*(*s*|*E*,*G*) factorizes into contributions from all connected pairs in the tree and the calculation becomes fully tractable, including all integrals over the potentials *E*, and even the sums over all possible trees *G* [29].

The DCA approach is very different in spirit from the “theoretically optimal” calculation sketched in eqs (1) and (2) above. Instead of comparing the probability of the alignment *S* under different possible contact graphs *G*, it assumes a model in which all pairs of positions interact to some extent and predicts contacts by sorting them by the inferred strength of the interaction. Specifically, it assumes the max-ent form that would be obtained by conditioning on pair-statistics

$$P_{\text{m}e}\left(s|\{E_{\alpha \beta }^{ij}\}\right)=\frac{1}{Z\left(\left\{E_{\alpha \beta }^{ij}\right\}\right)}\text{exp}[\left[\sum_{i<j}E_{s_{i}s_{j}}^{ij}\right]$$ (3)

where the sum is over all pairs of positions (*i*, *j*) and the $\left\{E_{\alpha \beta }^{ij}\right\}$ are potentials associated with the direct interactions between each pair of positions (*i*, *j*). These potentials are then fitted to the data, either by expansion around small energies [7] or using a pseudo-likelihood calculation [30]. Because the number of parameters $\left\{E_{\alpha \beta }^{ij}\right\}$ can still be large relative to the amount of data in the alignment *S*, additional regularization is used to avoid over-fitting, i.e., by using a prior $P(\{E_{\alpha \beta }^{ij}\})$ that favors energies of small absolute size. To predict contacts, the pairs (*i*, *j*) are then sorted by an ad hoc score quantifying the strength of the inferred direct interaction in terms of the fitted potential $\left\{E_{\alpha \beta }^{ij}\right\}$. In the original DCA [7], a mutual information was calculated for each pair (*i*, *j*) under the assumption that the dependence between *i* and *j* resulted only from the estimated direct energies $E_{\alpha \beta }^{ij}$. More recently, the norm $\sum_{\alpha ,\beta }{\left(E_{\alpha \beta }^{ij}\right)}^{2}$ has also been used, yielding equal or even better performance in tests [34].

In my personal opinion, the Bayesian network approach is attractive because it implements a straightforward simplification of the formal solution that probability theory prescribes, but it pays a huge price by restricting to tree structures, which we know is not realistic for protein structures. The max-ent form Eq (3), while not as general as the full Markov random field model (1), is clearly a much more powerful model. However, the way contact predictions have so far been extracted using this model is rather ad hoc and does not correspond to a methodologically sound application of probability theory. That is, instead of assuming only some pairs interact directly and attempting to infer which, the DCA approach rather assumes all pairs interact and attempts to rank the pairs by the strength of their interaction. This raises the question of whether it would be possible to use the more powerful model model (3) in a way that is a closer approximation to the calculation that probability theory prescribes. I want to end by making some suggestions in this direction.

One can start from the general Markov random field form Eq (1) and make the simplifying assumption that energies for all cliques of order higher than pairs are zero. That is, we only retain potentials *E*^*ij*^ for all edges (*i*, *j*) that occur in the contact graph *G*. Note that this is equivalent to the max-ent form Eq (3), but incorporates the additional constraint that potentials are zero for pairs that are not directly contacting in *G*. Formally, to calculate *P*(*S*|*G*) one would now marginalize over all possible potential functions *E*^*ij*^, i.e., performing the integrals Eq (2), but these integrals appear intractable. However, we could approximate these integrals by the value of the probability *P*(*S*|E_*_,*G*) at the set of potentials E_*_ that maximizes this likelihood. That is, we approximate *P*(*S*|*G*) by *P*(*S*|E_*_,*G*). It appears to me that it would be straightforward to adapt the pseudo-likelihood approach presented in [34] to calculate, for any possible graph *G*, the optimal pseudo-likelihood that can be obtained using the model (3), with the constraints $E_{\alpha \beta }^{ij}=0$ whenever (*i*, *j*) are not contacting in *G*. This also suggests a procedure for calculating scores for individual contacts (*i*, *j*). The highest possible likelihood *P*(*S*|E_*_,*G*) is of course obtained for the full graph *G*_*all*_ in which all pairs are allowed nonzero potentials. For each pair (*i*, *j*) one could calculate the maximum likelihood *P*(*S*|E_*_,*G*_*ij*_) that can be obtained using the graph *G*_*ij*_ that contains all edges except the edge (*i*, *j*) and use the likelihood ratio *P*(*S*|E_*_,*G*_*all*_)/*P*(*S*|E_*_,*G*_*ij*_) as a score for edge (*i*, *j*). It would be interesting to investigate if such calculations further improve the performance of contact predictions.

Finally, I want to close by mentioning one “elephant in the room” that has so far not been discussed. The sequences in our alignments *S* are related by common descent and all current approaches use a number of ad hoc “corrections” to minimize the effects of phylogenetic dependencies between the sequences. Moreover, it has been repeatedly demonstrated that these corrections are crucial for the performance of the methods. Given this, a more rigorous treatment of this phylogenetic signal would perhaps lead to the most significant improvement in contact prediction.
